# Granzyme B in peripheral blood mononuclear cells as a measure of cell-mediated immune response in paraneoplastic neurological syndromes and malignancy

**DOI:** 10.1007/s00262-020-02750-1

**Published:** 2020-11-02

**Authors:** Mikołaj Piotr Zaborowski, Patrycja Stefens-Stawna, Krystyna Osztynowicz, Tomasz Piorunek, Halina Batura-Gabryel, Agnieszka Dyzmann-Sroka, Wojciech Kozubski, Ewa Nowak-Markwitz, Sławomir Michalak

**Affiliations:** 1 Department of Gynecology, Obstetrics and Gynecologic Oncology, Division of Gynecologic Oncology, Poznan University of Medical Sciences, Poznań, Poland; 2 Department of Neurology, Poznan University of Medical Science, Poznań, Poland; 3 Department of Neurochemistry and Neuropathology, Chair of Neurology, Poznan University of Medical Sciences, Poznań, Poland; 4 Department of Pulmonology, Allergology and Respiratory Oncology, Poznan University of Medical Sciences, Poznań, Poland; 5 Cancer Registry, Greater Poland Cancer Centre, Poznań, Poland; 6 Department of Tumor Pathology and Prophylaxis, Poznan University of Medical Sciences, Poznań, Poland; 7 Neuroimmunological Diseases Unit, Polish Academy of Sciences, Warsaw, Poland

**Keywords:** Paraneoplastic neurological syndromes, Cytotoxic immune response, Granzyme B, Ovarian cancer, Lung cancer

## Abstract

**Background:**

Paraneoplastic neurological syndromes (PNS) may coexist with ovarian or lung cancers. Some tumors coexisting with PNS are smaller and have a better prognosis than tumors without PNS. PNS may constitute an opportunity to observe a natural immune antitumor response. We aimed to investigate a cytotoxic immune response by measuring granzyme B (GrB) in peripheral blood mononuclear cells (PBMC) in patients affected with ovarian or lung malignancy, with and without accompanying PNS.

**Methods:**

We enrolled patients with: nonmalignant lesions (*n* = 21), ovarian cancer (*n* = 19), lung cancer (*n* = 57), and PNS (*n* = 30). PBMC were isolated by density gradient centrifugation with Ficoll–Paque. We evaluated the expression of GrB in PBMC lysates by ELISA and normalized to protein content as measured by the Lowry method.

**Results:**

GrB levels in PBMC in the group with malignant tumors—median 1650 pg/mg protein (interquartile range 663–3260 pg/mg) and in patients with PNS—median 1890 pg/mg protein (range 1290–2640 pg/mg) was lower than in control group with nonmalignant lesions—median 5240 pg/mg protein (range 2160–7440 pg/mg), *p* = 0.0003 and *p* = 0.0038, respectively. The differences in GrB levels in PBMC between these groups were independent of epidemiological factors—age, sex, body mass index (BMI), and the number of immune cells, as confirmed by multiple regression analysis. Within the group of patients with malignancy and PNS, GrB levels in PBMC were elevated if onconeural antibodies were detected (2610; 2390–3700 pg/mg protein) as compared to patients without antibodies (1680; 970–1880 pg/mg protein, *p* = 0.035). GrB in PBMC was higher if the malignancy was diagnosed at the low (3060; 2120–5220 pg/mg protein) as compared to the high stage (1330; 348–2140, *p* = 0.00048). In patients with lung cancer, the expression of GrB in PBMC was lower (1430; 635–2660 pg/mg protein) than in the group with ovarian cancer (2580; 1730–3730, *p* = 0.02).

**Conclusion:**

The cytotoxic response measured in peripheral blood by GrB in PBMC is impaired both in the course of malignancy and PNS. Levels of GrB in PBMC were higher if onconeural antibodies were detected. Tracking reactive immune responses, such as GrB in PBMC may have diagnostic and monitoring value in malignancy and PNS.

**Electronic supplementary material:**

The online version of this article (10.1007/s00262-020-02750-1) contains supplementary material, which is available to authorized users.

## Introduction

An antitumor immune response plays a crucial role in defense against cancer. In particular, cytotoxic T lymphocytes (CTLs), and natural killer (NK) cells exert an antitumor effect by the elimination of cancer cells. Tumor-infiltrating lymphocytes (TILs) found in malignant ovarian tumors have been associated with better survival and lower recurrence rate [1–3]. Similarly, TILs appear in lung cancer [4], and their high proportion proved markedly associated with improved survival [5]. CTLs and NK cells induce cancer cell apoptosis by, among other mechanisms, releasing granules with granzyme B. This protein contributes both to the perforation of a target cell membranes and induction of apoptosis.

Both ovarian cancer and lung cancer may coexist with paraneoplastic neurological syndromes (PNS). PNS is defined as a pathology of the nervous system in patients affected with malignancy, excluding tumor infiltration, compression, or metastasis [6, 7]. One of the most common PNS, Hu-syndrome, may coexist with small cell lung carcinoma (SCLC). It often manifests as a subacute sensory neuronopathy associated with anti-Hu antigen antibodies detected in serum [8]. Ovarian cancer, in turn, may be complicated by subacute cerebellar degeneration that is often related to anti-Yo antibodies [9].

 The diagnosis of PNS may antedate the manifestation of malignancy [10]. Patients affected with PNS undergo detailed oncological diagnostic investigations that frequently result in the detection of a tumor at an early stage of development. Moreover, some neoplasms concomitant with PNS are smaller in volume, rarely metastasize, and have improved prognosis compared to malignancies without PNS [11]. Cases of tumor regression in patients affected with SCLC have been reported [12]. Some patients with PNS may present neurological symptoms without clinically identifiable tumor. Therefore, it has been postulated that PNS may yield a rare opportunity to observe a naturally effective antitumor response [11].

The prevailing view on the pathogenesis of PNS is that the immune system eliminates tumor and nervous tissue cells that share common antigens, known as onconeural antigens [13]. Indeed, antibodies against these antigens—referred to as onconeural antibodies—are detected in PNS patients' serum. These antibodies serve as a diagnostic tool but probably play little role in the pathogenic mechanism of disease [14]. In PNS, however, CTLs reactive against onconeural antigens were detected [15, 16]. The nervous tissue infiltrates found in patients with PNS consisted mainly of CD3^+^ and CD8^+^ lymphocytes, as well as less numerous CD4^+^ cells [17–19]. In the cerebrospinal fluid of PNS patients, white blood cells increased, with elevated CD8^+^ and CD4^+^ T lymphocytes, natural killer cells (NK), and especially B cells [20–23]. However, some studies failed to detect T lymphocytes reactive against onconeural antigens [24, 25]. Ovarian cancer tumors that developed in patients with anti-Yo paraneoplastic cerebellar degeneration were reported to have more infiltrates of T and B cells than ovarian cancer patients without PNS [26]. The increased CTLs activity could explain the small size of the tumors and improved prognosis in cancer coexistent with PNS.

The cytotoxicity potential of CTLs is reflected among other markers by the expression of granzyme B (GrB). GrB released into the extracellular space promotes cell death and inflammatory reaction [27]. The pro-apoptotic function is dependent on the presence of perforin [28, 29]. Granzymes are serine proteases that may induce cytochrome c release, directly activate caspases, or interfere with cell membrane integrity by cleavage of lamin B [27]. These are mainly cytotoxic T lymphocytes and NK cells in peripheral blood, in contrast to other CD4 + lymphocytes or neutrophils that produce GrB [30, 31].

Our study aimed to investigate the expression of GrB in peripheral blood mononuclear cells (PBMC), including CTLs and NK cells in patients with ovarian or lung cancer as well as with PNS. We hypothesized that GrB expression would (1) be decreased in both cancer types as a hallmark of an impaired antitumor response. We also anticipated that (2) in PNS patients, GrB expression would be elevated as a reflection of increased immune reactivity against malignancy and nervous tissue.

## Methods

### Patients

From 2010 to 2013, we enrolled 110 Caucasian patients in the study. 40 patients with histologically confirmed ovarian cancer (*n* = 19) and benign lesions (*n* = 21), including nonmalignant ovarian tumors (*n* = 12), leiomyomas (*n* = 8), and ascites unrelated to malignancy (*n* = 1) were recruited in Department of Gynecologic Oncology, Poznan University of Medical Sciences. Ovarian tumors were staged according to FIGO (International Federation of Gynecology and Obstetrics) system. We included 57 patients with lung cancer who were hospitalized in the Department of Pulmonology, Allergology, and Respiratory Oncology, Poznan University of Medical Sciences. Among lung cancer patients 20 (35%) were diagnosed as small-cell lung cancer, 12 (21%) as adenocarcinoma, 7 (12%) as squamous cell carcinoma, 3 (5%) as nonsmall cell lung cancer, and 15 (26%) as not otherwise specified (NOS). Lung tumors were staged following the American Joint Committee on Cancer (AJCC) TNM system. We also collected the samples from 30 patients with PNS admitted to the Department of Neurology, Poznan University of Medical Sciences. Some of those patients were previously hospitalized because of coexisting malignancy in the Department of Gynecologic Oncology or Department of Pulmonology, Allergology, and Respiratory Oncology, Poznan University of Medical Sciences. Patients with benign lesions were considered as a “Control” group (*n* = 21) and compared to 1) patients with PNS (*n* = 30) and 2) patients with “malignancy with no PNS” (*n* = 59). The PNS diagnosis was established according to clinical criteria published by Graus and co-authors, as summarized in Supplementary Table 1 [6]. PNS may affect both the central and peripheral nervous systems (Supplementary Table 2). The classical central nervous system PNS include encephalomyelitis, limbic encephalitis, subacute cerebellar degeneration, opsoclonus-myoclonus. The classical peripheral and neuromuscular PNS comprise subacute sensory neuronopathy (SSN), chronic gastrointestinal pseudo-obstruction, Lambert–Eaton myasthenic syndrome, and dermatomyositis. PNS may also manifest as nonclassical neurologic disorders, as described in detail by Graus and co-authors [6]. Based on the clinical presentation, the presence of a tumor, and onconeural antibodies' status, the syndromes are classified as "Definite" or "Possible" following Graus criteria (Table 1). We have always considered chemotherapy-induced polyneuropathy in the differential diagnosis. The neuropathies were considered as paraneoplastic only if the time of onset was not related to chemotherapy. Patients who had received chemotherapy before are specified in (Table 1). We identified the onconeural antibodies in peripheral blood (Supplementary Table 3). Additional studies, including magnetic resonance imaging (MRI), neurophysiological examination, or microscopic examination of cerebrospinal fluid, were performed as needed. We presented detailed clinical characteristics of PNS patients in (Table 1). All patients provided a signed informed consent, approved by the Ethics Review Board of Poznan University of Medical Sciences (Consent No 526/20 and 425/12).

**Table 1:** Clinical characteristics of patients affected with PNS

Paraneoplastic neurological syndromes									
Sex	Age	Neurological syndrome	Antibodies	Malignancy	Time from PNS onset to cancer diagnosis [months]		mRS baseline	mRS one year follow-up	PNS treatment
F	57	Subacute sensorimotor neuropathy	Anti-Ri, anti-myelin	Lung cancer*^, #^		( +) 12	2	3	IVIG
F	60	SSN	Seronegative	Lung cancer*		(−) 6	2	3	IVIG
M	65	SSN	Anti-Hu, anti-amphiphysin	Lung cancer*^, #^		(−) 4	1	1	Pregabalin
M	63	MND°	Seronegative	Lung cancer*^, #^		(−) 6	2	2	No PNS treatment
M	57	PCD	Seronegative	Lung cancer*^, #^		(−) 24	1	2	IVIG
F	56	SSN	Anti-Hu, anti-amphiphysin	Lung cancer*^, #^		(−) 1	4	4	IVIG
F	59	SSN	Anti-Hu	Lung cancer*^, #^		(−) 24	2	2	
M	57	PCD	Seronegative	Lung cancer*^, #^		( +) 1	1	1	IVIG
F	53	PCD	Seronegative	Lung cancer*^, #^		(−) 3	3	4	IVIG
M	71	PCD	Seronegative	Lung cancer^#^		(−) 6	2	3	IVIG
F	58	SSN°	Anti-MAG	Lung cancer^#^		(−) 24	1	1	Pregabalin
M	73	Subacute sensorimotor neuropathy	Anti-Ma/Ta, anti-Hu	Lung cancer		(−) 2	2	2	IVIG
M	68	SSN°	Anti-myelin	Lung cancer^#^		(−) 6	2	1	IVIG
M	71	PCD	Seronegative	Lung cancer		(−) 6	2	3	IVIG
F	74	MND°	Anti-MAG	Lung cancer^#^		(−) 6	2	2	IVIG
M	64	Subacute sensorimotor neuropathy°	Seronegative	Lung cancer		( +) 6	1	1	IVIG
F	72	Subacute sensorimotor neuropathy°	Seronegative	Lung cancer		(−) 24	1	1	IVIG
F	54	PCD, SSN	Seronegative	Lung cancer^#^		(−) 4	1	2	IVIG
F	51	Myasthenic syndrome	Anti-Hu, anti-myelin	Lung cancer		( +) 9	2	3	Plasma exchange
F	72	SSN	Anti-Hu	Lung cancer		(−) 3	1	0	IVIG
M	82	Subacute sensorimotor neuropathy°	Seronegative	Lung cancer^#^		(−) 4	2	1	Pregabalin
F	60	PCD	Anti-Ri, anti-amphiphysin, anti-Ma/Ta	Ovarian cancer^#^		(−) 8	2	2	IVIG
F	62	SSN°	Seronegative	Ovarian cancer^#^		(−) 3	2	1	Pregabalin
F	59	PCD	Seronegative	Ovarian cancer		(−) 6	3	2	IVIG
F	48	SSN	Anti-amphiphysin	Ovarian cancer^#^		(−) 3	2	1	IVIG
F	62	PCD	Anti-Yo	Ovarian cancer^#^		(−) 1	3	3	IVIG
M	76	MND, SSN	Seronegative	Prostate cancer		(−) 6	3	6	IVIG
M	86	SSN	Seronegative	Prostate cancer		( +) 3	1	1	Pregabalin
F	57	SSN	Anti-Yo	No tumor		N/A	2	1	Pregabalin
M	73	Myopathy, subacute sensorimotor neuropathy	Anti-Ma/Ta, anti-Ri, anti-amphiphysin, anti-myelin	No tumor		N/A	1	1	Pregabalin

In the column “Time from PNS onset to cancer diagnosis [months]” (−)—indicates that PNS preceded malignancy diagnosis, ( +)—indicates that PNS symptoms developed after cancer diagnosis

*MND* motor neuron disease, *PCD* paraneoplastic cerebellar degeneration (subacute cerebellar degeneration), *SSN* subacute sensory neuronopathy

*Small cell lung cancer, ^#^previous treatment with chemotherapy, °‘Possible’ PNS (based on Graus criteria) [6], *mRS* the Modified Rankin Scale, *N/A* not applicable

### Blood samples

**Fig. 1:**
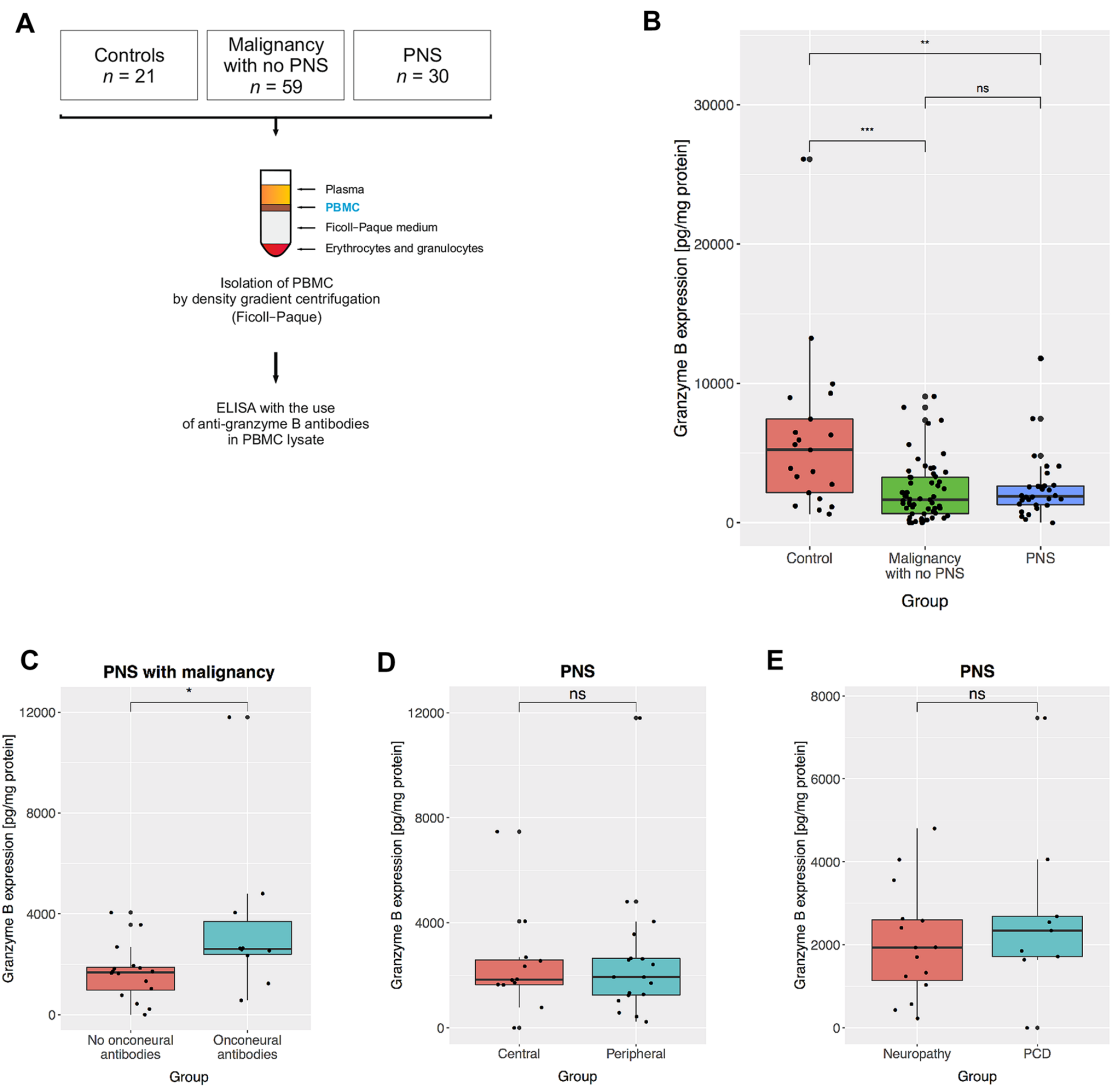
Granzyme B in peripheral blood mononuclear cells (GrB-PBMC) in malignancy and paraneoplastic neurological syndromes (PNS). **a** Schematic of an experimental procedure to isolate PBMC and measure GrB-PBMC in study groups. **b** GrB-PBMC in patients with nonmalignant lesions (nonmalignant ovarian tumors and leiomyomas), malignant tumors (collectively ovarian and lung), and PNS. **c** GrB-PBMC in patients with PNS with and without onconeural antibodies detected in serum. **d** GrB-PBMC in patients with PNS that clinically affected either the peripheral or central nervous system. **e** GrB-PBMC in patients with paraneoplastic peripheral neuropathy and paraneoplastic cerebellar degeneration (PCD). The central line corresponds to the median. The upper and lower “hinges” correspond to the first and third quartiles. *P* values are denoted as asterisks: ns—*p* > 0.05, *p < 0.05; **p < 0.01; ***p < 0.001, ****p < 0.0001. A statistical test is specified in the text

Samples of peripheral blood were collected by venipuncture (Fig. 1a). Serum was then aliquoted and frozen at − 80 °C for antibody analysis. Peripheral blood mononuclear cells (PBMC) were isolated within 1 h after collection by density gradient centrifugation with Ficoll–Paque (GE Healthcare). The onconeural antibodies were detected through indirect immunofluorescence (Euroimmun, cat. No FA-1111-1005-8) and confirmatory Line Blot (Euroimmun, cat. No DL 1111-1601-7 G) performed according to the manufacturer’s protocol. Our protocol enabled us to search for antibodies against the following antigens: amphiphysin, CV2, PNMA2 (Ma-2/Ta), Ri, Yo, Hu, recoverin, SOX1, titin, Zic4, GAD65, Tr, MAG, myelin. Our Department of Neurochemistry and Neuropathology participates on a regular basis in the external quality control of onconeural antibodies, superficial anti-neuronal antibodies, and anti-neural antibodies (e.g., anti-gangliosides) analyses. It received certification from Institut für Qualitätssicherung Lübeck. The measurements of ovarian tumor biomarker CA125 and HE4 were performed in serum by electro-chemiluminescence immunoassay (Roche) following manufacturer’s procedure.

### Granzyme B expression analysis

Granzyme B expression was measured in PBMC (referred to as GrB-PBMC). PBMC were lysed in buffer (150 mM NaCl, 50 mM Tris–HCl, 5 mM EDTA, and 1% vol/vol Triton X-100, pH = 8.0.) containing proteinase inhibitors (Sigma-Aldrich, cat. No P8340). The expression of GrB was detected in the supernatant employing ELISA with the use of anti-granzyme B antibodies (Abcam, cat. No ab46142). The results were normalized to the protein content in a sample and expressed in pg/mg protein. The estimation of protein content was performed by the Lowry method [32].

### Statistics

The analysis of data was performed in the R programming language (version 3.4.1) using RStudio (version 0.98.1060). The normality of distribution was verified with the Shapiro test. Groups with normal distribution were compared with the *t*-Student test (*t* test). Groups with distribution deviating from normal were compared using the Mann–Whitney test (wilcox.test). Values of the median or mean and interquartile range are given for each group. R packages (ggplot2, ggpubr) were used to generate plots (geom_boxplot() function) and add labels with applied statistics and *P* values (stat_compare_means() function). The central line in the plots corresponds to the median. The upper and lower “hinges” correspond to the first and third quartiles. Plot whiskers extend to the most extreme data point, which is within 1.5 times the interquartile range from the box. The results were considered significant for *P* values < 0.05. *P* values were either specified in the figure or denoted as asterisks: ns—*p* > 0.05, *p < 0.05; **p < 0.01; ***p < 0.001, ****p < 0.0001. We determined linear relationships between parameters utilizing the Pearson and Spearman correlation coefficients for normal and non-gaussian data distribution, respectively. We investigated the impact of possible confounding factors by multiple regression models using the lm() function.

## Results

### Granzyme B in PBMC in malignancy and PNS

The expression of GrB-PBMC in patients with malignant tumors (1650; 663–3260 pg/mg protein, median, interquartile range) was reduced as compared to the group with nonmalignant lesions (5240; 2160–7440 pg/mg protein, *p* = 0.0003, Mann–Whitney test, Fig. 1b). In PNS, GrB-PBMC expression was also reduced (1890; 1290–2640 pg/mg protein) in comparison to patients with nonmalignant lesions (5240; 2160–7440 pg/mg protein, *p* = 0.0038, Mann–Whitney test, Fig. 1b). Although we initially hypothesized that PNS might contribute to the more aggressive antitumor response, patients affected with PNS did not differ from those with malignancy in terms of GrB-PBMC (Fig. 1b). We also looked into the relationship between cellular and antibody-mediated immune response. Within the group of patients with malignancy and PNS the expression of GrB-PBMC was elevated if onconeural antibodies were detected (2610; 2390–3700 pg/mg protein) as compared to patients without antibodies (1680; 970–1880 pg/mg protein, *p* = 0.035, Mann–Whitney test, Fig. 1c). Although analyzing clinical presentation of PNS, we observed there was no difference between patients affected with peripheral (1940; 1250–2640 pg/mg protein) and central (1840; 1650 – 2580 pg/mg protein) nervous system syndromes (*p* = 0.98, Mann–Whitney test, Fig. 1d). In particular, GrB-PBMC was comparable between individuals suffering from peripheral neuropathy and PCD (mean 2031.5 *vs*. 2701.8 pg/mg protein, *p* = 0.40, *t*-Student test, Fig. 1e).

GrB-PBMC could be potentially affected by age, sex, and body mass index (BMI) (Table 2). To test whether those factors contribute to the differences in GrB-PBMC, we fitted a multiple regression model of GrB-PBMC values that included groups (“control”, “malignancy with no PNS” and “PNS”), age, sex, and BMI as possible covariates. Group assignment appeared the only factor affecting GrB-PBMC (*p* = 0.002 for “malignancy with no PNS” and *p* = 0.009 for “PNS” group, Table 3) after adjusting for age, sex, and BMI (*p* = 0.521, *p* = 0.278, *p* = 0.653, respectively). One could also hypothesize that GrB-PBMC is dependent on the number of immune cells. It is worth noting that to minimize the effect of differences in mononuclear cell counts, we normalized GrB measurements to total protein, which is proportional to the number of input cells. In the multiple regression model (Supplementary Table 4), the group assignment turned out to be the only valid factor affecting GrB-PBMC values (*p* = 0.0001 for”malignancy with no PNS” and *p* = 0.004 for “PNS” group) after controlling for absolute lymphocyte count (*p* = 0.227), percentage of lymphocytes in the WBC (*p* = 0.968), absolute monocyte count (*p* = 0.783), or percentage of monocytes in the WBC (*p* = 0.728). Taken together, GrB-PBMC is lower in patients with malignancy and in the group with PNS as compared to the control group irrespectively of epidemiological factors and immune cell counts.

**Table 2:** Epidemiological characteristics and parameters of the complete blood count

Group	Control	Malignancy with no PNS	PNS
Number of patients	21	59	30
Age [years]	50 (45–54)	58.5 (51.2–65.8)	62 (57–71.8)
BMI [kg/m^2^]	25.8 (24–31.2)	26.8 (24.7–31)	25.1 (22.2–27.7)
Absolute lymphocyte count [G/L]	1.6 (1.29–1.95)	1.54 (1.09–2.09)	1.65 (1.14–2.07)
Percentage of lymphocytes in the WBC [%]	24.6 (16.5–29.6)	21.6 (14.7–30.2)	29.1 (18.8–34.3)
Absolute monocyte count [G/L]	0.54 (0.42–0.72)	0.6 (0.42–0.81)	0.5 (0.41–0.635)
Percentage of monocytes in the WBC [%]	7.1 (5.7–8.8)	8 (6.3–9.25)	8.04 (7.35–10.1)

Values represent medians and interquartile ranges. *WBC* white blood cells, *BMI* body mass index, *PNS* paraneoplastic neurological syndromes

**Table 3:** Multiple regression analysis of GrB-PBMC values in the model, including Group ("control", "malignancy with no PNS" and "PNS"), age, sex, and BMI as possible covariates

Variable	GrB-PBMC [pg/mg]		
	*β*	SE	*P* value
Group—Malignancy with no PNS	− 3344.59	1036.34	0.00185**
Group—PNS	− 3168.99	1182.32	0.00901**
Age [years]	23.82	36.95	0.52104
Sex—Male	− 1155.82	1058.40	0.27826
BMI [kg/m^2^]	33.43	73.99	0.65269

*BMI* body mass index, *PNS* paraneoplastic neurological syndrome, *β* regression coefficient, *SE* standard error, **< 0.01

### Granzyme B in PBMC in ovarian tumors

**Fig. 2:**
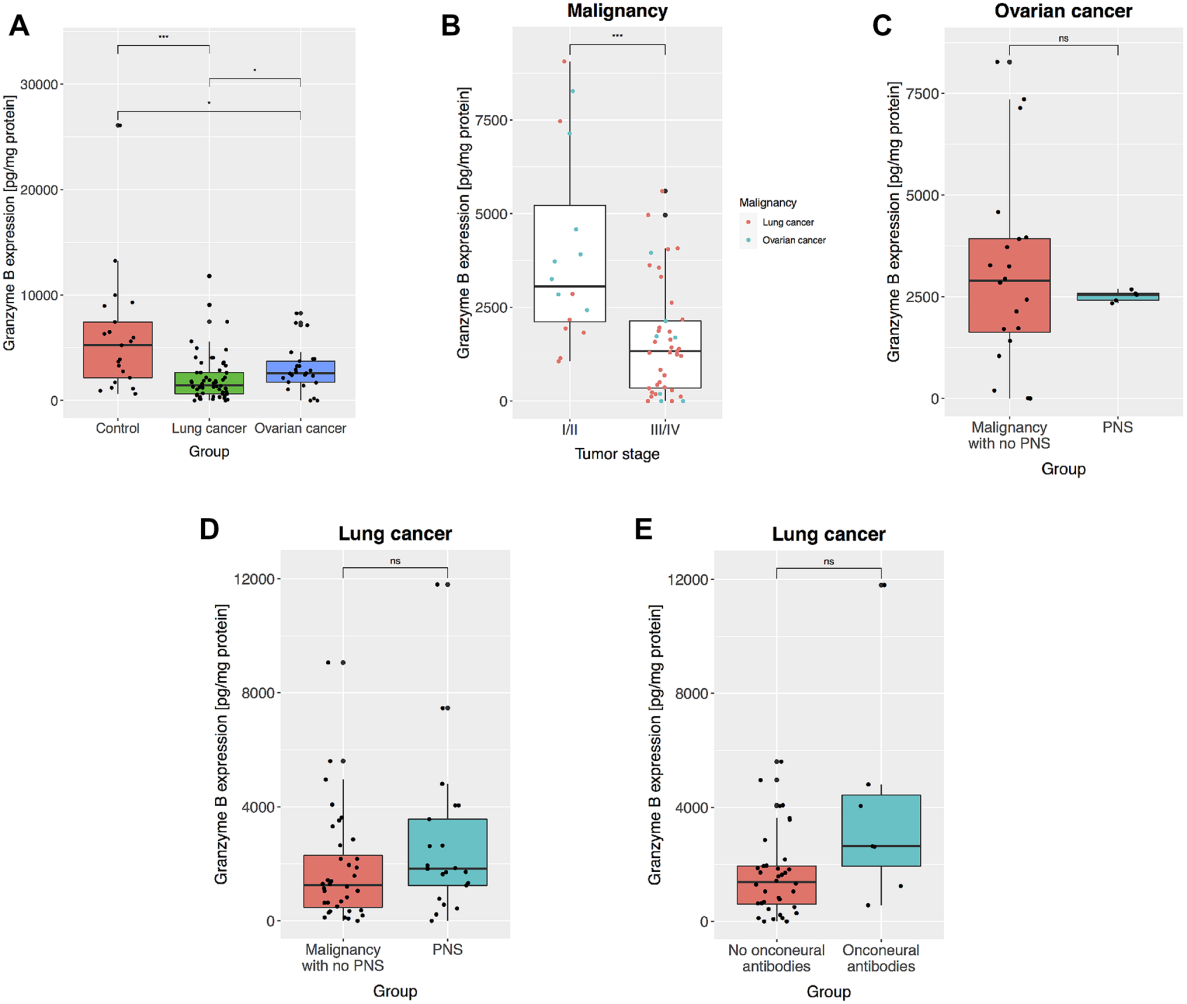
Granzyme B in peripheral blood mononuclear cells (GrB-PBMC) in ovarian and lung tumors. **a** The expression of GrB-PBMC was compared between patients with nonmalignant lesions (ovarian tumors and leiomyomas), lung cancer, and ovarian cancer. **b** GrB-PBMC in ovarian and lung cancer depending on the stage of malignancy. Ovarian tumors were staged according to FIGO (International Federation of Gynecology and Obstetrics) system. Lung tumors were staged following the American Joint Committee on Cancer (AJCC) TNM system. **c** GrB-PBMC in ovarian cancer coexisting with PNS and without PNS. **d** GrB-PBMC in lung cancer coexisting with PNS and without PNS. **e** GrB-PBMC in lung cancer with and without onconeural antibodies. The central line corresponds to the median. The upper and lower “hinges” correspond to the first and third quartiles. *P* values are denoted as asterisks: ns—*p* > 0.05, *p < 0.05; **p < 0.01; ***p < 0.001, ****p < 0.0001. A statistical test is specified in the text

The expression of GrB-PBMC was lower in ovarian cancer (2580; 1730–3730 pg/mg protein; median; interquartile range) than in benign lesions (5240; 2160–7440 pg/mg protein, *p* = 0.04, Mann–Whitney test, Fig. 2a). GrB-PBMC level was not associated with ovarian cancer biomarkers CA125 (*r* =—0.3, *p* = 0.08, Spearman test) or HE4 (*r* =− 0.23, *p* = 0.20, Spearman’s test) expression. One could hypothesize that the effectiveness of antitumor immune response was dependent on the stage of the disease. We have observed that patients affected with a more advanced stage of ovarian cancer—FIGO III had lower expression of GrB-PBMC than those with less severe disease—FIGO I or II (mean 1387 *vs*. 4513 pg/mg protein, respectively, *p* = 0.01, *t*-Student test). Patients with ovarian cancer stage FIGO I or II (3730; 3050–5530 pg/mg protein), however, did not differ from the control group (5240; 2160–7440 pg/mg protein) in terms of GrB-PBMC expression (*p* = 0.8765, Mann–Whitney test). We noticed that both lung and ovarian tumors had higher GrB-PBMC values if the malignancy was diagnosed at the low (3060; 2120–5220 pg/mg protein) as compared to the high stage (1330; 348–2140, *p* = 0.00048, Mann–Whitney test, Fig. 2b). These relationships will need to be confirmed in a larger group of patients. There was no difference in GrB-PBMC between ovarian cancer patients with PNS and without PNS (mean 2515 *vs*. 3095 pg/mg protein, *t*-Student test, *p* = 0.28, Fig. 2c).

### Granzyme B in PBMC in lung cancer

In patients with lung cancer the expression of GrB-PBMC was lower (1430; 635–2660 pg/mg protein) than in patients with benign lesions (5240; 2160–7440 pg/mg protein, *p* = 0.0001, Mann–Whitney test, Fig. 2a). We also observed it was reduced as compared to patients with ovarian cancer (2580; 1730–3730, *p* = 0.02, Mann–Whitney test, Fig. 2a). There were no differences in GrB-PBMC expression between lung cancer patients with PNS (1820; 1240–3560 pg/mg protein) and without PNS (1250; 472–2300 pg/mg protein, *p* = 0.14, Mann–Whitney test, Fig. 2d). In lung cancer, the expression of GrB-PBMC was not different between patients with onconeural antibodies (2640; 1940–4430 pg/mg protein) and in patients without antibodies identified (1380; 603–1950 pg/mg protein, *p* = 0.052, Mann–Whitney test, Fig. 2e). Given PNS appears especially in patients with small-cell lung cancer (SCLC), we analyzed this group separately. We found, however, no difference in GrB-PBMC between lung cancer patients with SCLC (1718, 1001–2289 pg/mg protein) and non-SCLC histology (1394, 383–3204 pg/mg protein, Mann–Whitney test, *p* = 0.4720).

## Discussion

We have shown decreased GrB-PBMC in patients with malignant ovarian and lung tumors compared to patients with nonmalignant lesions (Figs. 1b and 2a). This observation could be explained by a dysfunction of cytotoxic cells—a state potentially promoted by the cancer microenvironment. The reduced activity of peripheral blood lymphocytes (PBL) could correspond to the impaired potential of lymphocytes in the tumor. The degranulation of GrB takes part in the elimination of ovarian cancer cells by NK cells [33]. High expression of GrB and specifically high GrB/Foxp3 ratio in ovarian cancer tissue after the neoadjuvant chemotherapy was reported in patients with favorable progression-free survival [34]. Inhibition of GrB-mediated cytotoxicity could provide a growth advantage for cancer cells. It is hence possible that low GrB-PBMC reflects an even more reduced level of this protein in tumor tissue as a result of the immunosuppressive microenvironment. An impairment of cytotoxic immune response may also result from the phenomenon of exhaustion of lymphocytes [35]. Immune cells are highly active at the early stages of the host response to malignancy. However, they become exhausted after a prolonged exposure to tumor antigens, which results in a decreased production of GrB and other cytotoxic molecules [35]. That would be in line with our observation that GrB levels were even lower in patients with more advanced (III/IV) than early-stage (I/II) malignancy (Fig. 2b). However, we cannot be sure that a low GrB-PBMC directly reflects reduced GrB expression in the tumor microenvironment. An analysis of paired tumor tissue and PBMC samples could address this point in a future study. Taken together, the decrease of GrB-PBMC in the course of malignancy that becomes even more severe in patients with advanced-stage cancer may suggest that GrB-PBMC is an adequate measure of dysfunction of cytotoxic cells.

We have demonstrated that GrB-PBMC also drops in patients affected with PNS. Previous studies reported the existence of cytotoxic lymphocytes active against onconeural antigens. The analysis of cell specificity in anti-Hu syndrome revealed the existence of cytotoxic T lymphocytes aggressive towards HuD protein [16]. This antigen was found both in cells within SCLC tumors and in neurons. The tumor-infiltrating lymphocytes were also shown to react specifically with HuD antigen [36]. Interestingly, patients afflicted with SCLC-associated anti-Hu syndrome with high pleocytosis turned out to have a better overall survival rate than those with low pleocytosis [22]. Those reports were in agreement with the effective antitumor response in the course of PNS. On the other hand, some studies failed to detect HuD-specific T cells in peripheral blood of patients with the anti-Hu syndrome. Following the T cell stimulation with dendritic cells and HuD peptides, de Jongste and co-authors found no HuD-specific T cells, while applying sensitive techniques, including tetramer staining, detection of the type 2 cytokines (IL-4, IL-5, and IL-13) and INF-gamma secreting cells [25]. Similarly, HuD-specific T cells were not identified in cerebrospinal fluid in patients with anti-Hu syndrome [24]. Our initial hypothesis assumed that GrB-PBMC would increase in PNS as compared to patients with malignancy. Our data, however, indicate that the GrB-PBMC decrease in PNS, which mimics the profiles observed in malignancy (Fig. 1b). Neither in ovarian nor in lung cancer was the coexistence of PNS related to increased cytotoxicity as measured by GrB-PBMC (Fig. 2c, d). A host cytotoxic response may be effective at the beginning of original tumor development, but in the long run, a phenomenon of exhaustion may affect lymphocytes also in PNS. Indeed, two distinct functional forms of cytotoxic lymphocytes were described in the Hu-syndrome associated with SCLC. In the acute phase of PNS, there were specific immune cells releasing type 1 cytokines (TNF, Il-6, and Il-17), which promoted cytotoxic response. In the chronic phase of PNS, however, lymphocytes produced more type 2 cytokines (Il-4, Il-5, and Il-13) that abated antitumor cytotoxic reaction. This functional transition was likely related to cytokines released by the SCLC tumor [37]. Thus, it can be hypothesized that the immunosuppressive molecules released by a growing tumor impair an initially aggressive cell-mediated immune response that emerges at the beginning of PNS. Cancer cells may release immunosuppressive mediators, which inhibit overall GrB expression in tissue infiltrating lymphocytes and PBMCs. In that sense, GrB could be a measure of immune suppression induced by growing tumors and in the course of PNS. Interestingly, we demonstrate that GrB-PBMC was higher in PNS patients with identified onconeural antibodies (Fig. 1c). That could suggest that a subgroup with both increased GrB-PBMC and detectable onconeural antibodies represent PNS mediated by autoimmune mechanisms. In contrast, patients with low GrB-PBMC and no onconeural antibodies could be PNS that developed in an alternative nonimmune process. That is in line with some previous studies that failed to detect signs of the immune response in nervous tissue in some patients or animal models with PNS [38–40]. It is unlikely that the distinction between these subgroups comes from heterogeneity in clinical presentation since we observed no difference between PNS affecting the peripheral and central nervous system (Fig. 1d, e). Higher GrB-PBMC values in patients with onconeural antibodies do not prove the autoimmune mechanism—it is also possible they reflect a group of patients with less advanced tumors. This hypothesis remains to be verified in a future study. We also need to consider the possibility that only a subset of lymphocytes, for instance, Hu antigen-specific cells, are more cytotoxic, which does not translate into an overall increased activity of all lymphocytes. Since some of the previous studies failed to detect HuD-specific T lymphocytes [24, 25] in peripheral blood and cerebrospinal fluid, it is also plausible that reactive cytotoxic cells are only present locally—in the tumor microenvironment and affected nervous tissue. To sum up, our results suggest that cell-mediated immune response measured in peripheral blood is reduced in PNS to a similar extent as in cancer.

GrB increases intracellularly in activated CTLs and NK cells [41]. Granzymes, however, can also be released into the extracellular space [27]. Some studies have assessed serum levels of GrB in cancer patients [42]. We have previously demonstrated that GrB measured directly in serum (not in PBMC) was not different among patients affected with ovarian cancer, endometrial cancer, and endometriosis [43]. Other authors who studied angina pectoris also tried to detect GrB in plasma; however, it was detectable only in a subset of patients [44]. For this reason, they cultured PBMC from the patients and measured GrB in the conditioned medium, which revealed differences between stable and unstable angina pectoris patients. Of note, they observed similar differences when analyzing the expression of GrB in CD3 + lymphocytes as measured directly by flow cytometry [44]. Taken together, both our previous results and other studies imply the informativeness of measuring GrB in PBMC. However, developing methods for future studies might require a side-by-side comparison of GrB-PBMC and GrB measured in serum/plasma from the same patients. The low intracellular GrB-PBMC could relate to higher serum levels due to the release of GrB from cells.

It is worth noting there is evidence of the role played by tumor-derived neutrophils, which express GrB in antitumor activity mediated by Toll-like receptor 4 agonist [45]. Thus, the very early antitumor immune response that involves GrB can be mediated by neutrophils in the tumor microenvironment, and the PBMC reaction might be secondary.

In our study, we included three patients affected by MND. We are aware that the paraneoplastic origin of MND can be disputable. MND is not included in the "classical" clinical manifestations associated with paraneoplastic neurological syndromes. However, even a "definite" diagnosis can be made when well-characterized onconeural antibodies are detected or when neurological improvement is observed after cancer treatment. The diagnosis of MND in those three patients was established within 6 months before cancer detection. MRI of the brain and spinal cord revealed no lesions. All patients manifested the lower motor neuron syndrome in upper limbs and one mixed with the upper motor syndrome in lower limbs. No well-defined onconeural antibodies were detected, but two patients were clinically stable during oncological treatment without worsening, which is expectable in "pure" neurodegenerative MND. One patient died due to pulmonary embolism. Our decision to include those patients is supported by the study by Mele et al. (2018), in which the authors analyze eight original cases, and 21 patients identified from a systematic review of the literature [46]. Mele et al. proposed following features of paraneoplastic MND: subacute onset, lower motor neuron syndrome, associated or not with upper motor neuron involvement, predominant asymmetric upper limb involvement, presence of other nonmotor neurological manifestations, including sensory neuronopathy; signs of inflammation in the cerebrospinal fluid; neurological improvement or stabilization after immunotherapy and tumor treatment.

One patient was diagnosed with myopathy due to muscle weakness, manifested proximally without skin changes, but with increased serum creatine kinase activity (850 U/L). No muscle biopsy was performed; thus, there was limited data for polymyositis diagnosis, and therefore we assigned the symptoms to "myopathy". Moreover, the patient manifested subacute sensorimotor neuropathy, whose onset was associated with muscle symptoms. We considered the paraneoplastic origin because three well-defined onconeural antibodies' coexistence was detected (anti-Ma/Ta, anti-Ri, and anti-amphiphysin).

We did not assign the patient with the myasthenic syndrome to Lambert–Eaton syndrome. She clinically manifested proximal muscle weakness but no autonomic dysfunction. Reduced compound motor action potentials (CMAPs) and decrements were found on EMG, revealing postsynaptic block. We did not perform a test for anti-VGCC antibodies; however, we detected no anti-SOX antibodies. Therefore, we used more general term “myasthenic syndrome” instead of LEMS.

The coexistence of two or more well-defined onconeural antibodies is considered a strong indicator of cancer, even if it does not manifest clinically. Pittock (2005) reported multiple antibodies in 31% of analyzed samples [47]. We are aware that anti-myelin and anti-MAG antibodies in line with anti-nucleosome antibodies detected using commercial kits are non-onconeural. The clinical significance of such unspecific antibodies is not clear. However, the association of anti-MAG antibodies with neuropathy is already established. We mentioned those antibodies' presence to show that nonspecific autoimmune reactions can be associated with some tumors.

Our study has several potential clinical applications. (1) GrB-PBMC seems to be a good measure of immune suppression in the course of malignancy. It may still be challenging to establish a diagnosis of malignant ovarian or lung tumors before surgery. An assessment of reactive immune dysfunction status as measured by GrB-PBMC could identify patients at higher risk of malignancy and help in the differential diagnosis. Measuring the activity of peripheral blood lymphocytes with a different cytotoxicity assay proved informative for the risk of future cancer incidence [48]. The decline in GrB-PBMC might be an additional indicator of relapse in patients during follow-up after chemotherapy completion. (2) Tracking of immune suppression in the course of diagnosed ovarian cancer could help to personalize therapy by identifying patients potentially responsive to immunotherapies [49]. (3) Measurement of GrB-PBMC could also be useful in the differential diagnosis of patients affected with PNS. One would expect an increase in GrB activity in autoimmune disorders. PNS without a clear diagnosis of a tumor may present clinically as an autoimmune disease. A drop in GrB-PBMC might suggest underlying PNS rather than other autoimmune pathologies.

Our study demonstrated that the function of PBMCs measured by the expression of GrB is impaired both in the course of malignancy and PNS. We observed that this dysfunction became even more pronounced in patients with more advanced ovarian cancer. These observations may suggest that GrB levels measured in peripheral blood cells are responsive to local pathology involving tumor and paraneoplastic reaction. Therefore, tracking reactive immune responses, as measured by GrB-PBMC, may be both useful in diagnostic procedures and informative regarding the course of malignancy and PNS.

## Electronic supplementary material

Below is the link to the electronic supplementary material.

Supplementary file1 (DOCX 109 kb)
