# Supplementary material for: Granzyme B in peripheral blood mononuclear cells as a measure of cell-mediated immune response in paraneoplastic neurological syndromes and malignancy
Source: Cancer Immunol Immunother. 2020 Nov 2;70(5):1277–89. doi: 10.1007/s00262-020-02750-1 (PMC8053162; doi:10.1007/s00262-020-02750-1)
Supplement: Supplementary file 1 — Supplementary file1 (DOCX 109 kb) [file 262_2020_2750_MOESM1_ESM.docx]

**Supplementary Table 1.** Definite and possible PNS according to diagnostic criteria as published by Graus and co-authors [6]

| **Diagnostic criteria for diagnosis of PNS** | |
| --- | --- |
| Definite PNS | Possible PNS |
| 1. A classical syndrome and cancer that develops within five years of the diagnosis of the neurological disorder.  2. A non-classical syndrome that resolves or significantly improves after cancer treatment without concomitant immunotherapy, provided that the syndrome is not susceptible to spontaneous remission.  3. A non-classical syndrome with onconeural antibodies (well characterized or not) and cancer that develops within five years of the diagnosis of the neurological disorder.  4. A neurological syndrome (classical or not) with well characterized onconeural antibodies (anti-Hu, Yo, CV2, Ri, Ma2, or amphiphysin) and no cancer. | 1. A classical syndrome, no onconeural antibodies, no cancer is diagnosed, but at high risk for having an underlying tumor.  2. A neurological syndrome (classical or not) with partially characterized onconeural antibodies and no cancer.  3. A non-classical syndrome, no onconeural antibodies, and cancer present within two years of diagnosis. |

**Supplementary Table 2.** Clinical manifestation of paraneoplastic neurological syndromes and malignancies. n – number of patients, MND - motor neuron disease, PCD - paraneoplastic cerebellar degeneration, SSN - subacute sensory neuronopathy

| **Paraneoplastic neurological syndromes and tumor location** | | |  |  |
| --- | --- | --- | --- | --- |
| **Tumor location** | Ovarian cancer  *n* = 5 | Lung cancer  *n* = 21 | Prostate cancer  *n* = 2 | No malignancy  *n* = 2 |
| **Clinical presentation of PNS** | PCD (*n* = 3),  SSN (*n* = 2) | subacute sensorimotor neuropathy (*n* = 5),  SSN (*n* = 7),  PCD (*n* = 5),  PCD and SSN (*n* = 1),  myasthenic syndrome (*n* = 1), MND (*n* = 2) | SSN (*n* = 1),  SSN and MND (*n* = 1) | SSN (*n* = 1),  myopathy and subacute sensorimotor neuropathy (*n* = 1) |

**Supplementary Table 3.** Onconeural antibodies identified in patients with paraneoplastic neurological syndromes and malignancies. n – number of patients. * – non-well characterized onconeural antibodies

| **Onconeural antibodies in PNS and tumor location** | | |  |  |
| --- | --- | --- | --- | --- |
| **Tumor location** | Ovarian cancer  *n* = 5 | Lung cancer  *n* = 21 | Prostate cancer  *n* = 2 | No malignancy  *n* = 2 |
| **Antibodies** | anti-Yo (*n* = 1),  anti-amphiphysin (*n* = 1),  anti-Ri + anti-amphiphysin + anti-Ma/Ta (*n* = 1),  seronegative (*n* = 2) | anti-Hu (*n* = 2),  anti-Ri + anti-myelin^*^ (*n* = 1), anti-MAG^*^ (*n* = 2), anti-Ma/Ta + anti-Hu (*n* = 1), anti-Hu + anti-amphiphysin (*n* = 2),  anti-Hu + anti-myelin^*^ (*n* = 1),  anti-myelin^*^ (*n* = 1),  seronegative (*n* = 11) | seronegative (*n* = 2) | anti-Yo (*n* = 1),  anti-Ma/Ta + anti-Ri + anti-amphiphysin + anti-myelin^*^  (*n* = 1) |

**Supplementary Table 4.** Multiple regression analysis of GrB-PBMC values in the model including Group ("control", "malignancy with no PNS" and "PNS"), absolute lymphocyte count, percentage of lymphocytes in the WBC, absolute monocyte count and percentage of monocytes in the WBC. WBC – white blood cells, PNS – paraneoplastic neurological syndrome, *β* – regression coefficient, SE – standard error, ** - <0.01, *** < 0.001

| Variable | GrB-PBMC [pg/mg] | | |
| --- | --- | --- | --- |
|  | ***β*** | **SE** | ***P-*value** |
| Group - Malignancy with no PNS | -3699.576 | 923.949 | 0.000141 *** |
| Group - PNS | -3340.323 | 1118.844 | 0.003781 ** |
| Absolute lymphocyte count [G/L] | 859.525 | 705.160 | 0.226553 |
| Percentage of lymphocytes in the WBC [%] | 1.966 | 49.178 | 0.968217 |
| Absolute monocyte count [G/L] | -549.421 | 1989.488 | 0.783154 |
| Percentage of monocytes in the WBC [%] | -57.059 | 163.600 | 0.728202 |
